# Restructuring consciousness –the psychedelic state in light of integrated information theory

**DOI:** 10.3389/fnhum.2015.00346

**Published:** 2015-06-12

**Authors:** Andrew R. Gallimore

**Affiliations:** Computational Neuroscience Unit, Okinawa Institute of Science and Technology Graduate UniversityOkinawa, Japan

**Keywords:** integrated information theory, psychedelic state, psilocybin, ketamine, LSD, neural complexity, integration, neural entropy

## Abstract

The psychological state elicited by the classic psychedelics drugs, such as LSD and psilocybin, is one of the most fascinating and yet least understood states of consciousness. However, with the advent of modern functional neuroimaging techniques, the effect of these drugs on neural activity is now being revealed, although many of the varied phenomenological features of the psychedelic state remain challenging to explain. Integrated information theory (IIT) is one of the foremost contemporary theories of consciousness, providing a mathematical formalization of both the quantity and quality of conscious experience. This theory can be applied to all known states of consciousness, including the psychedelic state. Using the results of functional neuroimaging data on the psychedelic state, the effects of psychedelic drugs on both the level and structure of consciousness can be explained in terms of the conceptual framework of IIT. This new IIT-based model of the psychedelic state provides an explanation for many of its phenomenological features, including unconstrained cognition, alterations in the structure and meaning of concepts and a sense of expanded awareness. This model also suggests that whilst cognitive flexibility, creativity, and imagination are enhanced during the psychedelic state, this occurs at the expense of cause-effect information, as well as degrading the brain's ability to organize, categorize, and differentiate the constituents of conscious experience. Furthermore, the model generates specific predictions that can be tested using a combination of functional imaging techniques, as has been applied to the study of levels of consciousness during anesthesia and following brain injury.

## Introduction

The psychological state elicited by the classic psychedelics drugs, such as LSD and psilocybin, is one of the most fascinating and yet least understood states of consciousness. Although biochemical and pharmacological studies of these drugs are detailed and extensive (Nichols, 2004), until recently, studies using human subjects have been limited. However, with the advent of modern functional neuroimaging techniques and the successful maneuvering around mulish political obstacles, the neural correlates of the psychedelic state in humans are now being revealed. This has prompted the development of new models of the effects of these drugs on neural function and their relationship to the phenomenological features of the psychedelic state (Vollenweider et al., 1997; Carhart-Harris et al., 2012, 2014).

Integrated information theory (IIT) is one of the foremost contemporary theories of consciousness (Oizumi et al., 2014). By mathematically formalizing the properties of subjective consciousness self-evident to humans, IIT attempts to explain the conditions under which systems, such as the brain, are conscious and for the phenomenological structure of conscious experience. Although IIT is continually under development, it is now a mature and rigorous theory that considers both the quantity and quality of consciousness in all its usual states, including waking, REM, and NREM sleep, during seizures and under anesthesia. As such, it would be extremely surprising if IIT had nothing to inform us about the nature of the psychedelic state. As we appear to be within a growing resurgence of psychedelic research in humans, it seems entirely appropriate and timely to analyze the psychedelic state from the perspective of IIT, with the aim of providing fresh insights into this beguiling state of consciousness. Furthermore, the mathematical rigor with which IIT is constructed might allow any such insights to be similarly formalized, perhaps pointing toward a more quantitative measure of the psychedelic state and an explanation for the range of effects of different psychedelic drugs. Importantly, the most recent models of the psychedelic state, developed based on functional imaging data (Carhart-Harris et al., 2014), will be shown not only to be compatible with IIT, but complemented by it. This analysis will also generate testable predictions regarding phenomenological features of the psychedelic state and its neural correlates, and suggest specific experiments to guide further research.

## Phenomenology of the psychedelic state

The effects of psychedelic drugs on consciousness, particularly at higher doses, can be both profound and varied (Masters, 1966). However, the effects can loosely be placed into three categories:

Perceptual effects: altered shapes and colors (often appearing brighter, more vivid, and intense); visual distortions; visual hallucinations (open eye visuals and closed eye visuals); illusions; difficulty focusing; synaesthesias.
Psychic effects: mood alterations (from ecstatic euphoria to panic); time distortion; thought alterations (difficulty concentrating, strange concepts, ideas, or connections, increases in creativity); dreamlike feelings; depersonalization; the sense that awareness is “expanded”.
Somatic effects: dizziness; tremors; nausea; drowsiness; blurred vision. (Masters, 1966; Hollister, 1984; Nichols, 2004).

The relative prominence of particular effects can be strongly dependent on the specific drug, the dosage and the setting in which the drug is used. As such, it is beyond the scope of this discussion to attempt an explanation for all the varying phenomenological features of the psychedelic state elicited by different psychedelic drugs. However, recent functional imaging studies suggest that, at relatively low doses of the classic psychedelic psilocybin, the psychedelic state is characterized by an unconstrained form of cognition (Carhart-Harris et al., 2012, 2013; Roseman et al., 2014); the stream of conscious experience appears more fluid and dynamic, with novel neural states being explored (Tagliazucchi et al., 2014). It is straightforward to extrapolate this data to account for many of the perceptual and psychic effects that often manifest in the psychedelic state, although a more thorough and definitive treatment must await further human studies.

## Functional neuroimaging of the psychedelic state

The classic psychedelic drugs are all partial agonists at the serotonin 5HT_2A_ receptor, and this site has been established as the major locus for their effects (Vollenweider et al., 1998; Nichols, 2004). The earliest functional imaging studies suggested that psychedelic drugs, psilocybin specifically, are cortical activators and this was deemed to be consistent with the rich, heightened state of awareness characteristic of the psychedelic state (Vollenweider et al., 1997). Further, activation of 5HT_2A_ receptors is known to depolarize cortical pyramidal neurons. As such, it was something of a surprise, and ostensibly paradoxical, when a groundbreaking functional magnetic resonance imaging (fMRI) study with psilocybin revealed a global decrease in cortical activation (Carhart-Harris et al., 2012). However, recent magnetoencephalography (MEG) studies localized the activated pyramidal neurons to the deep layer V of the cortex, which provide predominantly inhibitory feedback, explaining why activation of this neural population results in a decrease in neural activity (Muthukumaraswamy et al., 2013). The conflict with earlier data has been explained as an effect of the difference in temporal resolution between positron emission tomography (PET) and fMRI. It should be noted that, from the perspective of IIT, neural activation holds no primacy over deactivation, as both can be equally informative.

Maximal decreases in activation were observed in cortical “hubs,” such as the anterior and posterior cingulate cortices (ACC and PCC) and the thalamus, as well as the medial prefrontal cortex (mPFC). Further, the intensity of the subjective effects of the drugs correlated with the magnitude of this deactivation (Carhart-Harris et al., 2012). Functional connectivity analysis revealed an uncoupling of activity between the mPFC and the PCC, both key components of the default mode network (DMN), indicating a loss of network integrity (Carhart-Harris et al., 2013; Muthukumaraswamy et al., 2013). The DMN, also known as the task negative network (TNN), is preferentially activated during introspection, daydreaming and memory retrieval (Qin and Northoff, 2011). Activation of this network is normally anti-correlated (inversely coupled) with activation of task positive networks (TPNs) (Fox et al., 2005), which are activated during attention-demanding tasks, such as attending to external visual stimuli. However, both fMRI and MEG revealed a decrease in the anti-correlation between the DMN and several TPNs, with an increase in the functional connectivity between these networks post-psilocybin. Overall, this functional imaging data indicates that psilocybin reduces the differentiation between brain networks and produces a “disorganized” brain state. This prompted Carhart-Harris and colleagues to propose that the psychedelic state is characterized by elevated neural entropy—the so-called “entropic brain” theory (Carhart-Harris et al., 2014).

According to this model, during normal waking consciousness, the brain operates at a slightly subcritical state between a highly ordered, low entropy (sub-critical) state and a highly disordered, high entropy (super-critical) one (Chialvo, 2010). This optimal position between perfect order and complete disorder allows the brain to operate in a well-defined and organized manner whilst maintaining a degree of pliability, which is essential for flexible and adaptable cognition. Psychedelic drugs, such as psilocybin, increase the entropy of the brain and generate a more disordered and fluid state of consciousness, as revealed by the functional connectivity analyses. Further, more targeted analyses of the psilocybin fMRI data showed an increase in the variance of neural activation across voxels within networks associated with higher cognition, indicative of more erratic neural activity. A formal measure of Shannon entropy was also employed and, in line with the predictions of the entropic brain theory, entropy was indeed elevated in these networks (Carhart-Harris et al., 2014).

A related approach was used to examine the entropy of the dynamic temporal sequence of states (discretized as functional connectivity motifs) explored by the brain post-psilocybin. Crucially, this analysis not only revealed that the sequence of states was more random (higher entropy) post-psilocybin, but also that specific connectivity motifs were exclusive to the psilocybin state (Carhart-Harris et al., 2014; Tagliazucchi et al., 2014). This indicates that psilocybin increases both the entropy of the brain and the size of its state repertoire, which will be of central importance when considering the psychedelic state from the perspective of IIT.

## Overview of integrated information theory (IIT)

Before examining the psychedelic state from the perspective of IIT, it is essential that the key ideas of IIT be presented, particularly those that will be used in the sections that follow. However, it is not the aim of this section to provide a detailed and thorough account of IIT and the reader is directed to Giulio Tononi's papers explaining the theory (Oizumi et al., 2014). This section will serve merely as a simplified overview of IIT, with particular emphasis placed on those aspects of the theory most informative to the psychedelic state.

IIT provides a formal description of both the quantity of consciousness and its quality (structure), both of which might be affected by psychedelic drugs. In order for a system to be conscious, it must be both highly differentiated (informative) and integrated. This means the information generated by the system as a whole must be beyond that generated by its individual components. That is, it must be irreducible. IIT proposes that a set of neural elements (e.g., thalamocortical columns) with the special property of being a local maximum of integrated information, quantified by Φ (big phi), is conscious. This set of neural elements is known as a *maximally irreducible conceptual structure* (also known as a *complex)*. Only a complex exists from its own perspective (there is *something-it-is-like-to-be it* Nagel, 1974). All conceptual structures, whether maximally irreducible or not, are defined as systems of *concepts*. Although a concept has a formal definition in IIT, this is simply a formalization of the more familiar definition: an abstract idea or an object of thought consisting of all the essential or characteristic features of that object. This could refer to a concrete object, such as an apple or a chair, but might also refer to something more abstract, such as the color red or the concept of freedom or happiness. As concept theory is a major branch of both classical and contemporary philosophy, it is perhaps safest to opt for the simplest definition—“concepts are the constituents of thoughts” (Margolis and Laurence, 2014), although “constituents of conscious experience” is perhaps a more helpful definition here.

**Figure 1:**
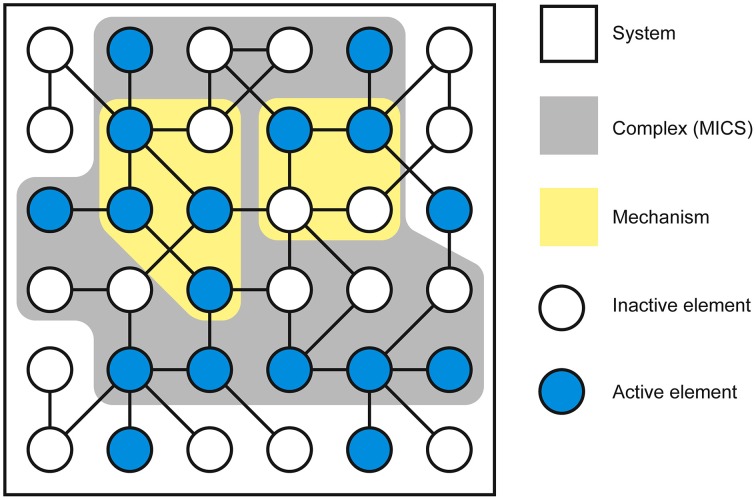
**Neural elements within the system form mechanisms (only 2 mechanisms within the power set are shown)**. The particular current state of the mechanism is defined by the activity or silence of individual neural elements and determined by their causal interactions. The complex comprises the set of neural elements in the system that forms a maximally irreducible conceptual structure (MICS).

**Figure 2:**
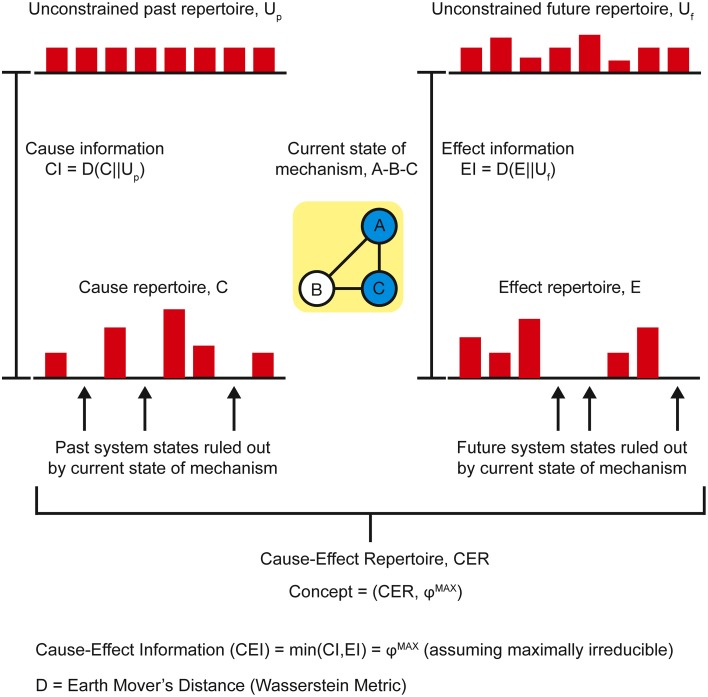
**The current state of a mechanism is associated with a specific cause-effect repertoire of past and future states of the system**. The mechanism generates information by ruling out certain past and future states, quantified as the distance between the cause-effect repertoire and the unconstrained past-future repertoire.

As conscious experience is built as a system of concepts, understanding the definition of a concept is central to understanding how IIT describes the structure of consciousness and how psychedelic drugs can change it. Assuming the cortical column is chosen as the basic unit of functional segregation, each column is an *element* of the *system* under consideration (e.g., a region of the cerebral cortex). Elements can be combined with other elements to form *mechanisms* (e.g., a set of directly or indirectly connected cortical columns engaging in some sort of causal interaction) (Figure 1). The element itself is the most elementary mechanism and elements can combine to form higher order mechanisms. For any set of elements, all possible mechanisms are represented as the *power set*, which includes all possible combinations of elements, including the highest order mechanism, which is the set itself. Dependent on the interactions between the elements of the mechanism and, in fact, with the elements of the entire system (in the case of the brain, the synaptic connections, their type, and weight, etc.), every mechanism occupies a particular state at any point in time (hereafter its *current state*). For example, particular sets of neurons might be firing, whilst others are quiet. As the system consists of causal, deterministic interactions, only certain system states could have *caused* the mechanism to enter its current state. This set of system states is a discrete probability distribution called the *cause repertoire* (IIT uses the term *repertoire* to describe various discrete distributions of system states). Likewise, given the current state of the mechanism, only a specific set of immediate future states will be available to the system—this is the *effect repertoire*. Both of these repertoires may be referred to together as the *cause-effect repertoire* (Figure 2). So, by entering its current state, a mechanism *constrains* both the past and future states of the system. In actuality, each individual mechanism may not constrain the past and future states of the entire system, but rather of a specific subset of elements called the *purview*. Any elements outside of the purview are unconstrained by the mechanism. However, all of the mechanisms together will constrain the past and future states of the entire system.

**Figure 3:**
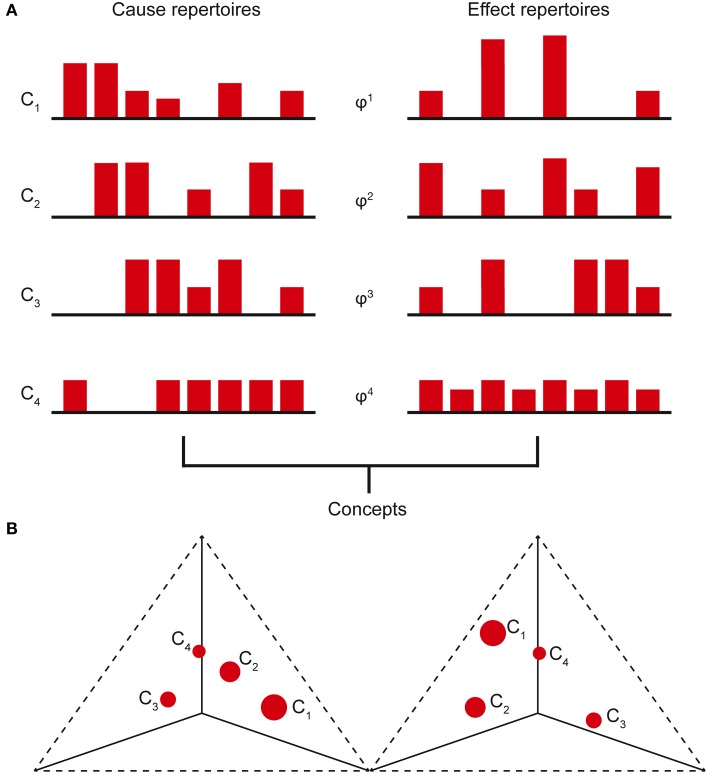
**(A)** The cause-effect repertoire of each mechanism within a complex, together with its associated φ value, constitutes a concept. The states of the cause-effect repertoire define what the concept “is about” and φ quantifies the degree to which the concept is present in the experience. **(B)** Concept (qualia) space is a high-dimensional space with each cause-effect repertoire state occupying a single axis. Plotting the concepts on these axes, the conceptual structure forms a shape in this space that is identified with the conscious experience (qualia). (Only 3 axes shown with past and future states plotted on separate axes).

Prior to considering the current state of any mechanisms, all the possible past and future states of the system form the maximal entropy *unconstrained past and future repertoires*, respectively (i.e., unconstrained by any mechanisms in any specific state). By entering a specific current state, a mechanism effectively rules out certain past and future system states (i.e., those past states that could not have caused the current state and those immediate future states that cannot follow) and thus generates information. The amount of information the mechanism specifies about the past state of the system is called the *cause information* and is obtained by calculating the [Earth Mover's] distance between the *cause repertoire* and the *unconstrained past repertoire*. The larger the difference between these distributions, the more the current state of the mechanism constrains the past states of the system and thus the more cause information is generated. If the cause information is zero (i.e., there is no difference between the distributions), the current state of the mechanism tells us nothing about the past state of the system. Likewise, the distance between the *effect repertoire* and the *unconstrained future repertoire* quantifies the *effect information*. The minimum of the cause and effect information is the *cause-effect information* (Figure 2). By systematically bipartitioning the mechanism, it is possible to calculate the *cause-effect information* specified by the whole mechanism above and beyond that specified by its parts. This is the integrated information specified by the mechanism and is known as φ (small phi). Overall, the elements of the mechanism, together with its cause-effect repertoire and associated value of φ, constitute a *concept* (Figure 3A). *The states of the cause-effect repertoire specify what the concept “is about” and φ quantifies the degree to which that concept is present within the conscious experience of the system*. Every possible mechanism may, in principle, specify a concept. However, if the mechanism fails to generate any integrated information (i.e., if φ = 0), then the concept does not exist from the subjective perspective of the complex (i.e., it is not present within the conscious experience).

Of all the concepts specified by the system, subsets of these can form *conceptual structures*, with each concept represented as a point in *concept space*, a high-dimensional space in which each possible past and future state within the cause-effect repertoire is represented by one dimension (axis). The coordinates of the point are given by the probabilities of each past and future state in the cause-effect repertoire and φ is represented by the size of the point. A conceptual structure thus forms a constellation of points in concept space (Figure 3B). Whilst each concept specifies a quantifiable amount of cause-effect information, a conceptual structure generates *conceptual information*, which is the systems equivalent of cause-effect information. The conceptual information of each concept is found by calculating the difference between the cause-effect repertoire of the concept and the unconstrained cause-effect repertoire (known as the *null concept*, a concept about nothing). This difference is then multiplied by the φ-value of the concept. Summing over all the concepts gives the conceptual information generated by the conceptual structure, denoted Φ (big phi). Clearly, a conceptual structure with many high-φ concepts that are well-separated from the null concept will generate a large amount of conceptual information. Conversely, a poorly integrated system with a limited repertoire of states will generate only a small amount of conceptual information (Oizumi et al., 2014).

In principle, any subset of concepts within the power set of concepts specified by the system can form a conceptual structure. However, only one conceptual structure can exist at any time from the intrinsic perspective of the system—this is the MICS (complex), which specifies the maximal amount of integrated conceptual information (Φ^max^, hereafter simply Φ). Not all concepts with non-zero φ will form part of this irreducible conceptual structure. This is intuitively obvious, as conscious experience changes from moment to moment, as concepts continuously enter and leave consciousness. Concepts that leave the complex cease to exist from the subjective perspective of the complex, but may re-enter the complex at a later time.

**Figure 4:**
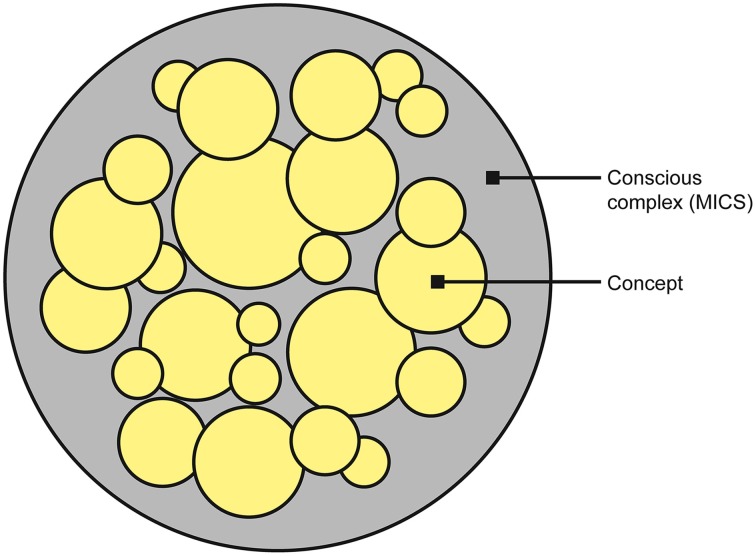
**The unified conscious complex (outer gray circle) is formed from a constellation of concepts (yellow circles)**. The degree to which each concept is present in the experience is quantified by φ (represented by the size of the circle), and the overall quantity of consciousness is quantified by Φ (represented by the size of the gray circle).

The subjective conscious experience of the complex is identified with the complex itself—the level or quantity of consciousness is identified with the value of Φ, whereas the constellation of concepts determines the structure or quality of the experience (quale) (Figure 4). Each concept contributes to the overall experience, with its cause-effect repertoire determining what that concept “is about” and its φ-value quantifying the degree to which the concept is present in the experience. Each concept is a point in a high-dimensional *qualia space* (this is the same as concept space, except only complexes (MICS) have qualia) and thus the constellation of concepts form a “shape” in this space (Balduzzi and Tononi, 2009). It is this shape that is identified with the quality of the experience. The higher the dimensionality of the qualia space, the richer the experience (i.e., the experience is “about more things” at once).

## The psychedelic state and IIT

IIT defines conscious experiences as a shape in qualia space. This applies to all conscious states, including the psychedelic state. To appreciate how IIT can provide an explanation for many phenomenological features of the psychedelic state, the following discussion will be separated into three sections. Firstly, using IIT and results from functional neuroimaging data, the effect of psychedelic drugs on neural differentiation (i.e., information) will be examined. Secondly, the effect of psychedelic drugs on neural integration will be addressed. Finally, experiments to test whether the psychedelic state is a state of increased consciousness (integrated information, Φ) will be suggested, together with predictions as to their outcome.

### The effect of psychedelic drugs on information

Functional neuroimaging data and its analysis support the proposal that psychedelic drugs increase the entropy of the brain (Carhart-Harris et al., 2014), producing a state of unconstrained cognition. More detailed analysis of this data revealed an increase in the repertoire of states explored by the brain, consistent with an increase in neural entropy (Tagliazucchi et al., 2014). From the perspective of IIT, both of these observations can be further elucidated, both at the level of the mechanism (concept) and at the level of the conceptual structure (complex).

Before discussing the effect of psychedelic drugs at the level of the concept, it is important to clarify exactly how information is defined within IIT. It is erroneous, from the perspective of IIT at least, to equate the size of a system's state repertoire (i.e., the number of states it can potentially occupy) with the information it generates. Although a large repertoire of states is necessary for a system to generate a large amount of information, it is not sufficient. Stated equivalently, increasing the size of the state repertoire does not necessarily imply an increase in information as defined by IIT. The amount of cause-effect information generated by a mechanism depends on the number of past and future states it *rules out* by entering its current state. Simply adding noise to a system by, for example, allowing neural elements to be randomly activated, will increase the state repertoire but will not increase information. This is because the current state of the system is unable to *constrain* random past or future states. Equivalently, the current state gives no information about those states that had no causal effect on it, nor about randomly generated future states.

Any IIT derived model of the psychedelic state must account for the increase in entropy and size of the state repertoire explored by the brain. In fact, this functional imaging data can be regarded as axiomatic and used as a foundation upon which to develop an IIT-based model of the psychedelic state. As IIT constructs conscious experience as a system of concepts, it makes most sense to begin at the level of the mechanism and then move to the level of the conceptual structure (complex).

**Figure 5:**
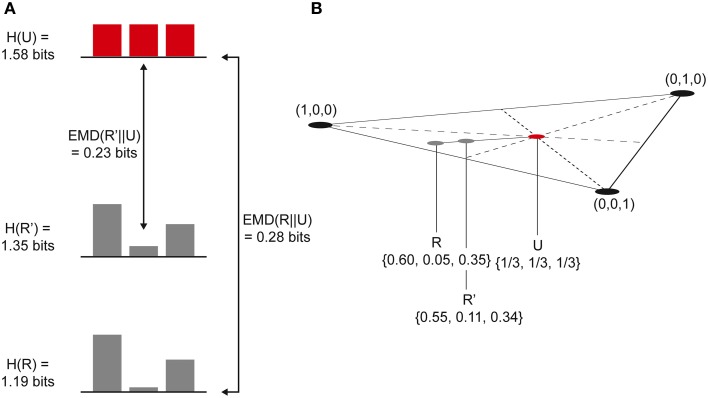
**(A)** Simple 3-state system. A psychedelic (cause/effect) repertoire, R', is generated using a linear combination of the (cause/effect) repertoire, R, and the unconstrained (past/future) repertoire, U. R' has a higher entropy than R and is closer to U, generating less (cause/effect) information. **(B)** Each repertoire can be plotted on a 3-simplex. The psychedelic repertoire, R', lies on a line between the (cause/effect) repertoire and the unconstrained repertoire.

When a mechanism enters a specific state, the cause and effect repertoires of system states are specified as distributions with lower entropy than the corresponding unconstrained past and future repertoires. The cause-effect information generated by the mechanism entering the current state is the minimum distance between these repertoires, as explained earlier. This provides a measure of how much the mechanism constrains past and future states of the system. The functional imaging data described earlier revealed that, post-psilocybin, the brain explores a larger repertoire of states and in a more random fashion. From the perspective of IIT, this suggests that the brain's past and future states are less constrained by the states of its mechanisms. As will be shown, and in agreement with this imaging data, this is equivalent to stating that the entropy of the cause and effect repertoires is increased. This means that the cause and effect repertoires of the mechanisms in the complex are shifted toward the unconstrained past and future repertoires. Mathematically, this can be modeled by taking a linear combination of the cause repertoire and the unconstrained past repertoire to generate a *psychedelic cause repertoire* (Figure 5A). A *psychedelic effect repertoire* can be similarly generated, by taking a linear combination of the effect repertoire and the unconstrained future repertoire. This approach takes into account the repertoire of states available to the system without invoking additional states resulting from random noise, and can be applied to both the cause repertoire and the effect repertoire to generate a *psychedelic cause-effect repertoire* for every mechanism in the complex (Note that this model does not explain *how* neural entropy is increased by psilocybin, but predicts consequences of the entropy increase revealed by functional imaging data).

If the unconstrained repertoire (past or future), *U*, contains n states, then *U* can be described as a point within an (n-1)-dimensional n-simplex. The cause or effect repertoire, *R*, is another point within this simplex and extending a line between *R* and *U* generates a range of repertoires moving from *R* to *U*, all of them linear combinations of *R* and *U* with increasing *U*-*character*. According to this model, the psychedelic repertoire, *R*′, lies somewhere on this *R–U* line (this is easier to understand using a diagram and is illustrated in Figure 5B for a system with only three possible states):

$$R^{\prime }=\left(1-t\right)R+tU$$

where *t* is the portion of *U* mixed with *R* and 0 ≤ *t* ≤ 1. Clearly, if *t* = 0, then *R*′ = *R*, and if *t* = 1, then *R*′ = *U*.

**Figure 6:**
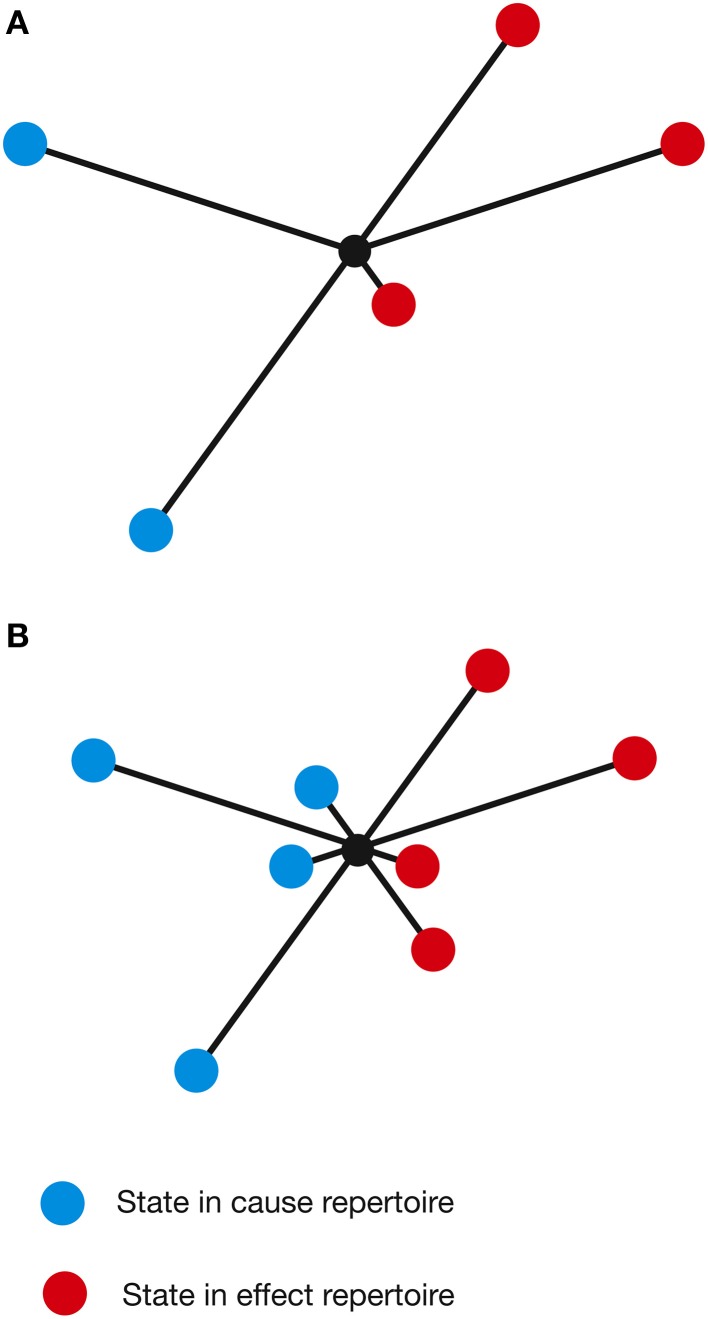
**A concept can be visualized by plotting the states of the cause-effect repertoire on a set of arbitrary axes, with the length of each line representing the probability of each state (φ not considered). (A)** A concept in a normal waking state. **(B)** A higher-dimensional psychedelic state concept, derived using the linear combination method described in the text.

As *U* is, by definition, the maximal entropy distribution, a linear combination of *U* and *R* will always generate a distribution with higher entropy than *R*. Furthermore, in order to move the cause-effect repertoire closer to these maximum entropy distributions, the probabilities of the individual states must be changed, such that formerly high probability states become lower probability and vice versa. Importantly, this is certain to include increasing the probability of certain states from zero or, at least, from a very low probability. In other words, new states would effectively be added to the cause-effect repertoire—the repertoire of potential system states, both past and future, is expanded (actually, the number of states does not increase, but certain states develop a non-zero probability). As this applies to all mechanisms in the complex, the brain would be observed to explore a larger number of states in a more random fashion. This is exactly what is observed in the functional imaging data. From a phenomenological perspective, this has important consequences for the structure of the concept itself. The states within the cause-effect repertoire define what the concept “is about” (Oizumi et al., 2014) and so an expansion of these states essentially changes the nature of the concept and what that concept means to the individual—the psychedelic concept becomes “about more things” or acquires additional characteristics not present in normal consciousness (Figure 6). It is interesting to speculate that this might account for the feeling that the world appears somewhat altered in the psychedelic state. Concepts that were once clear and well-defined become novel, strange and perhaps even bizarre or ludicrous.

Further insights are obtained by examining the effect of psychedelic drugs on the cause-effect information specified by each mechanism. The current presentation of IIT uses the Earth Mover's Distance (EMD, aka Wasserstein metric) to measure the distance between the distributions in calculating the cause-effect information (Oizumi et al., 2014). The EMD between the cause or effect repertoire, *R*, and the unconstrained repertoire, *U*, can be defined as follows:

If *X* and *Y* are random variables with distributions *R* and *U*, respectively, and *d(X,Y)* is a [Euclidean] distance metric, then the infimum of the expectation, E, taken over all possible joint distributions of *X* and *Y* (with *R* and *U* being the marginal distributions, respectively), gives the EMD:

EMD(R||U)= inf 𝔼[d(X,Y)]

Let X^*^ and Y^*^ be random variables with distributions *R* and *U*, respectively, which attain the infimum in the definition of the EMD, then:

$$EMD(R||U)=\;𝔼[d\left(X^{*},Y^{*}\right)]$$

*R* is combined with *U* using the method described above to generate a new distribution (the psychedelic cause/effect repertoire), *R*′:

$$R^{\prime }=\left(1-t\right)R+tU$$

where 0 ≤ *t* ≤ 1.

Now, to calculate *EMD*(*R*′||*U*) (i.e., the distance between the psychedelic repertoire, *R*′, and the unconstrained repertoire, *U*), we must define a new random variable, *X*', with distribution, *R*′, such that:

$$EMD(R^{\prime }||U)=inf\;𝔼[d\left(X^{\prime },Y^{*}\right)]$$

where,

$$X^{\prime }=\{\begin{array}{cc} X^{*}, & P(1-t)\\ Y^{*}, & P(t) \end{array}$$

Since *EMD*(*R*′||*U*) is, by definition, the infimum, it must always be the case that:

$$EMD(R^{\prime }||U)\leq 𝔼[d\left(X^{\prime },Y^{*}\right)]$$

Moreover:

$$𝔼\left[d\left(X^{\prime },Y^{*}\right)\right]=\left(1-t\right)𝔼\left[d\left(X^{*},Y^{*}\right)\right]+t𝔼[d\left(Y^{*},Y^{*}\right)]$$

And so, equivalently:

$$EMD(R^{\prime }||U)\leq \left(1-t\right)𝔼\left[d\left(X^{*},Y^{*}\right)\right]+t𝔼[d\left(Y^{*},Y^{*}\right)]$$

Since, plainly:

$$𝔼\left[d\left(Y^{*},Y^{*}\right)\right]=\;0$$

This simplifies to:

$$EMD(R^{\prime }||U)\leq \left(1-t\right)𝔼\left[d\left(X^{*},Y^{*}\right)\right]$$

As defined earlier:

$$EMD(R||U)=\;𝔼[d\left(X^{*},Y^{*}\right)]$$

So, substituting:

$$EMD(R^{\prime }||U)\leq \left(1-t\right)EMD(R||U)$$

And, since 0 ≤ (1 − *t*) ≤ 1, it follows that:

(1−t)EMD(R||U) ≤EMD(R||U)

And so:

$$EMD(R^{\prime }||U)\leq EMD(R||U)$$

So, increasing the entropy of the cause-effect repertoire will always lead to a reduction in its distance, as measured using the EMD, from the unconstrained past and future repertoires and hence a reduction in the cause-effect information specified by the mechanism.

Without considering any effect on neural integration, this result suggests a continuum of consciousness between the normal waking state and unconsciousness, with the psychedelic state lying somewhere in between, perhaps at varying distances from normal consciousness depending on the drug and dose. However, there is no reason to assume that the classic psychedelics are capable of producing such a maximal entropy state. Indeed, even high doses of psychedelics, such as psilocybin or LSD, are not accompanied by any appreciable loss of consciousness. The N-methyl-D-aspartate (NMDA) receptor antagonist, ketamine, is an unusual drug in that low to moderate doses have a definite psychedelic effect (Krystal et al., 1994; Gunduz-Bruce, 2009; Murray et al., 2013), whereas higher doses induce anesthesia. It is tempting to suggest that lower doses have a moderate effect on neural entropy, producing a psychedelic state in the same manner as psilocybin, for example, whereas higher doses cause a more dramatic increase in neural entropy that reduces cause-effect information such that Φ becomes minimal and consciousness is lost. Additionally, or alternatively, ketamine may have a more profound disruptive effect on neural integration than the classic psychedelics, as is seen with other anesthetic agents (Ferrarelli et al., 2010). These different effects ought to be distinguishable using functional imaging techniques.

This reduction in cause-effect information will also affect its ϕ-value; the degree to which the concept is present in the experience will be altered. It is tempting to insist that the concept's ϕ will necessarily decrease owing to a reduction in the cause-effect information. However, ϕ depends not only on the cause-effect information, but also on the degree to which this information is integrated. If psychedelic drugs act to increase integration across the cortex (discussed later), then it is possible that ϕ may actually increase. Further, it is possible that concepts with zero or very low ϕ, which don't exist to any significant extent during normal waking consciousness, may become present during the psychedelic state. As such, certain concepts experienced during the psychedelic state might simply not be available to someone who hasn't taken the drug and these concepts could have no relation to anything within the individual's normal conscious experience.

In summary, the effect of a psychedelic drug at the level of the concept is to increase the entropy of the cause-effect repertoire and, effectively, expand the number of available states within the repertoire, but reduce the cause-effect information specified by each mechanism as it enters its current state. This means that the mechanisms constrain past and future states of the brain less stringently; the current state of each mechanism is less informative about the brain's past and future states. This result agrees with imaging data showing that the brain explores an expanded repertoire of states in a more random fashion (Tagliazucchi et al., 2014), and resting state functional connectivity analysis suggesting networks become less differentiated (less anti-correlated) from each other in the psychedelic state (Roseman et al., 2014). As the DMN and TPNs are strongly anti-correlated during normal waking consciousness, states involving the DMN effectively “rule out” many states involving the TPNs, and vice versa, thus generating a large amount of information. The decrease in anti-correlation observed post-psilocybin means that DMN states are less effective at ruling out TPN states, thus reducing the information generated.

From the level of the concept, it is straightforward to extrapolate to the level of the conceptual structure and thus the quale. Changing both the cause-effect repertoire and ϕ-values of the concepts within the conceptual structure, and perhaps also incorporating additional concepts (i.e., those whose ϕ-value becomes non-zero), changes the shape of the structure in qualia space. Whether the integrated conceptual information, Φ, increases or decreases will depend on the effect on the individual concepts and how the psychedelic drug affects information integration (discussed later). However, recall that the dimensionality of qualia space is equal to the total number of states in the cause-effect repertoires of the constituent concepts. It is this dimensionality that determines the “richness” (Tononi, 2012) of the experience. Whilst it is not trivial to predict the effect on Φ (although suggestions for experiments to probe this issue will be discussed later), expanding the cause-effect repertoires of the concepts within the conceptual structure will generate a higher-dimensional quale that is experientially richer than normal waking consciousness—the psychedelic state is “about more things” than the normal waking state. The enhanced richness and expanded awareness of the psychedelic experience can thus be explained as an increase in the dimensionality of qualia space, rather than an increase in cortical activation. However, it is important to note that a “richer” conscious state does not necessarily imply an increase in the overall quantity of consciousness (quantified by Φ). In fact, as shown above, there is an inherent tension between cause-effect information and the dimensionality of the concepts and thus the quale. Despite this, cause-effect information is specified at the level of the concept, and it is conceivable, if integration is enhanced, that an increase in the number of concepts [with non-zero ϕ] could result in elevated Φ, even though the ϕ associated with individual concepts might fall. It isn't, therefore, a contradiction to suggest that both the richness and the quantity of consciousness might increase in the psychedelic state.

**Figure 7:**
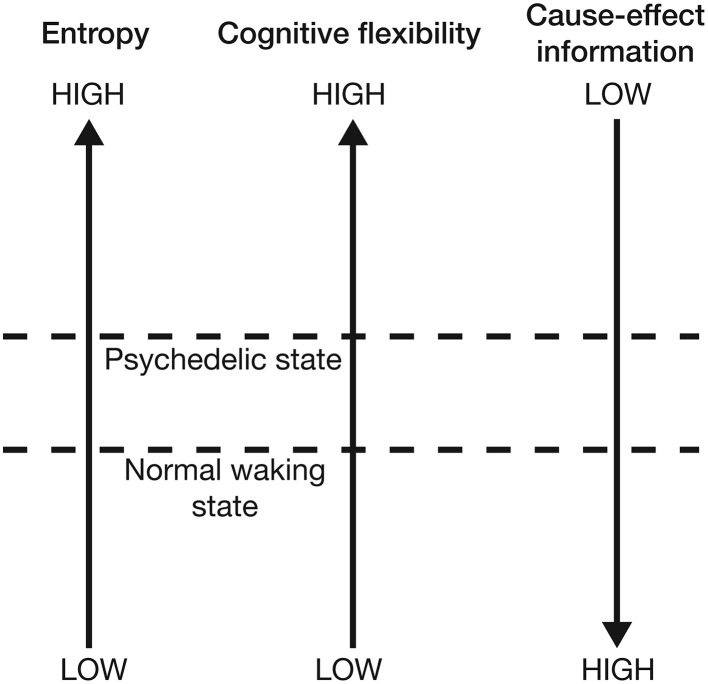
**Increasing neural entropy elevates cognitive flexibility at the expense of a decrease in the cause-effect information specified by individual mechanisms**.

**Figure 8:**
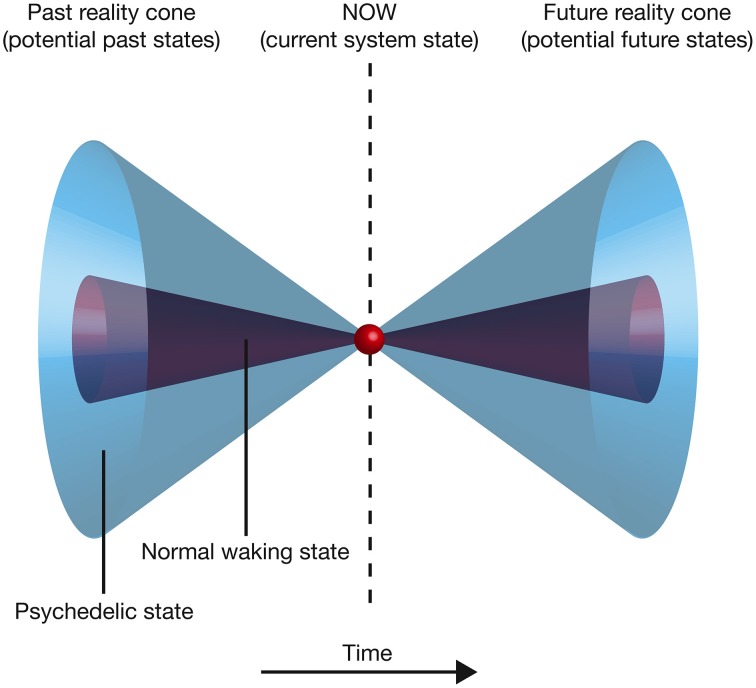
**Moving backward or forward in time from the current state of the complex, the number of potential states increases, producing a double “reality cone” extending into the past and the future**. The area of the circular section at each time point represents the number of potential states. Psychedelic drugs increase the number of potential past and future states, expanding the reality cones.

Overall, this model suggests that the expanded awareness and enhancement of cognitive flexibility afforded by the psychedelic drugs comes at a functional cost: increasing the entropy of the cause-effect repertoires of mechanisms within a complex expands the characteristics and meaning of concepts and produces a richer, more flexible state of consciousness (a greater range of brain states can be explored), but this is at the expense of the cause-effect information associated with each concept. This provides an elegant explanation for the need to optimize neural entropy—a very low entropy (highly ordered) state would maximally constrain past and future states of the brain and thus maximize cause-effect information. However, this would collapse the number of states within the cause-effect repertoire of each mechanism and thus produce a highly inflexible state of consciousness with a rigid sequence of conscious states. At the opposite extreme, excessively high entropy would maximize the number of states within the cause-effect repertoire of each mechanism, producing a highly flexible state of consciousness with the potential for creative and novel cognition. However, the cause-effect information specified by each mechanism would be very low; the mechanisms would fail to constrain the past and future states of the brain. If entropy is optimal, cause-effect information is high whilst a degree of cognitive flexibility is retained. Psychedelic drugs push the brain into a slightly higher entropy state, which is experientially richer and more flexible, but not as informative as normal waking consciousness (Figure 7). An intuitive way of appreciating this reduction in information is to consider that entropy is also a measure of uncertainty. High entropy states are less informative because they contain a greater uncertainty about the past states that caused the current state, and those states that will follow it. This potential for novel (and uncertain) state pathways makes it more difficult to make sense of the progression of conscious moments, and can be visualized by projecting forwards and backwards in time from the current state of the system. As one moves further from the current time, the number of potential system states increases. This generates a double “reality cone” projecting outwards from the current moment, representing the “spotlight” of consciousness into the past and future. By increasing the number of potential neural states, psychedelic drugs expand these reality cones, broadening the spotlight, but perhaps at the expense of focusing attention, organizing thoughts, and maintaining cognitive control (Figure 8).

**Figure 9:**
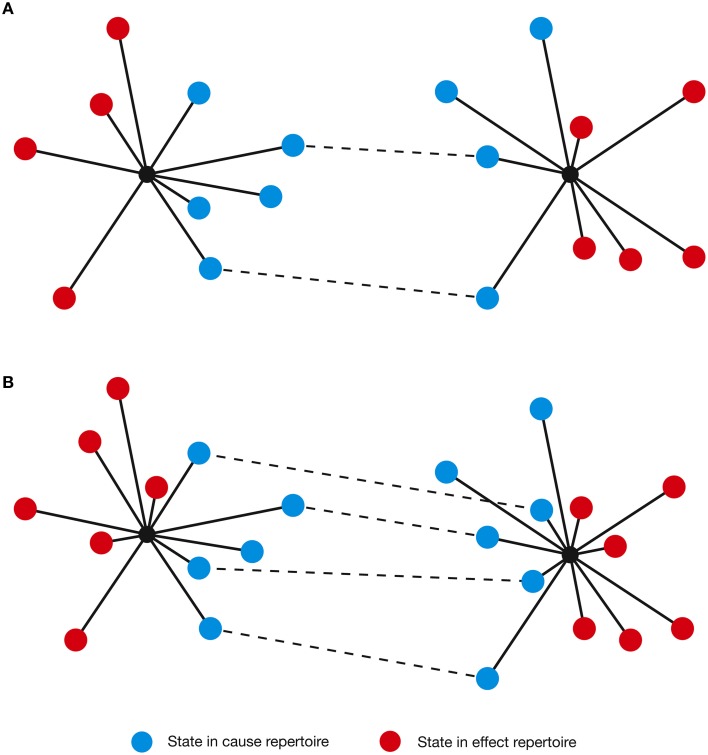
**(A)** A pair of concepts with a degree of conceptual overlap (shared states [in cause repertoire only for clarity] are indicated by dashed lines). **(B)** In the psychedelic state, the concepts increase in dimensionality, acquiring additional states. This increases the degree of conceptual overlap (i.e., the number of shared states).

By considering how psychedelics restructure concepts (and thus consciousness), it is possible to view the functional cost, and potential benefits, of psychedelics from a different perspective. We can use the example of two concepts in normal waking consciousness (Figure 9A). Although each concept forms a distinct shape in concept space, it is possible that they share a non-zero number of states. Some degree of *conceptual overlap* makes perfect sense, since not all concepts are independent. To use a trivial but concrete example from everyday life, the concept of an *apple* is not independent of the concept of an *orange*: they differ in enough characteristics such that they can be distinguished (e.g., color and texture), but they share enough that they can be connected and categorized (e.g., sweet, edible fruits). As demonstrated, the cause-effect repertoire representing a psychedelic concept possesses additional states not present in normal consciousness (Figure 9B). As these additional characteristics are not necessarily systematically acquired, but through an increase in entropy, conceptual overlap between certain concepts will inevitably increase, potentially even between those that are not normally related. This might explain why creativity, imagination, and novel thinking are enhanced during the psychedelic state (Sessa, 2008; Jones et al., 2009; Frecska et al., 2012), but it is straightforward to see how this could degrade the brain's ability to maintain the organization and differentiation of the concepts within conscious experience—an obviously essential adaptive skill. Small degrees of conceptual overlap could progress to a more outright *conceptual blending*, as the boundaries between concepts are dissolved, and might explain why Albert Hofmann, during the first ever LSD trip, was startled to observe:

*“The lady next door, whom I scarcely recognized… was no longer Mrs. R., but rather a malevolent, insidious witch with a colored mask.”*(Hofmann, 1980)

This experience could be interpreted as the conceptual blending of the concept of “Mrs. R” with the concept of a “malevolent witch.” Although these are not fully independent concepts, meaning they have a degree of conceptual overlap (e.g., both are female, and certainly they shared additional characteristics not revealed in the text), they are held distinct in normal consciousness. As the concept of “Mrs. R” acquired additional characteristics in Hofmann's psychedelic state, conceptual overlap may have increased between these concepts, such that Hofmann failed to distinguish between them: from his perspective, Mrs. R effectively *became* the malevolent witch. It is also possible that conceptual blending could result in a failure to distinguish concepts related to *self* from concepts related to *other*, perhaps explaining the “ego dissolution” (Carhart-Harris et al., 2012; Muthukumaraswamy et al., 2013) and the sense of “oceanic boundlessness” (Vollenweider, 2001) that are often feature in high-dose psychedelic experiences.

Carhart-Harris et al. (2014) suggest that neural entropy must be suppressed to allow the brain to “organize and constrain cognition.” This IIT-based model reaches an equivalent conclusion: entropy is suppressed to maximize cause-effect information and maintain the organization, categorization, and differentiation of concepts, which is essential for adaptively interacting with the world. However, this certainly doesn't mean that the psychedelic state of consciousness is without value. By expanding the reality cone and promoting conceptual overlap, the high entropy state facilitates creativity, novel thinking, and imagination, which are obviously of great importance in human cognition. Furthermore, psychedelics are beginning to show great promise as psychotherapeutic agents in the treatment of depression, anxiety, and obsessive compulsive disorder (Moreno and Delgado, 1997; Berman et al., 2000; aan het Rot et al., 2010; Buchborn et al., 2014), perhaps by allowing sufferers to break out of the inflexible and circular modes of thinking that characterize these conditions (Carhart-Harris et al., 2014).

### The effect of psychedelic drugs on neural integration

So far, only the effects of psychedelic drugs on neural differentiation has been considered and it has been implicitly assumed that the set of neural elements that constitutes the maximally integrated conceptual structure (complex) are unchanged by the drug. However, this is certainly an unreasonable assumption. At any point in time there should be a dominant complex of high Φ that comprises a specific set of neural elements across the cortex. However, even during normal waking consciousness, this is likely to be highly dynamic, with the composition of the complex changing from moment to moment, as certain subsets of neural elements are excluded from, or brought into, the complex (Oizumi et al., 2014). The phenomenology of the psychedelic state, which can include synesthesia and expanded awareness, might suggest that the integrated complex is more expansive during the psychedelic state, encompassing more of the cortex (i.e., more neural elements within the complex), but more empirical measures of integration are required.

Neural integration is commonly measured by the synchronization of neural activation –functional connectivity analysis using fMRI and coherence analysis to assess oscillatory synchrony using EEG or MEG. However, it is important to distinguish between functional and effective connectivity. Functional connectivity refers to *the temporal correlations between spatially remote neurophysiological events* (Friston, 1994), but provides no direct information about the origin of these correlations. Effective connectivity describes the causal influence of neural systems over each other (Friston, 1994). Once functional connectivity has been observed, measures of effective connectivity must be used to establish the nature of the causal interactions between neural systems (i.e., the effective connectivity); functional connectivity alone does not necessarily imply effective connectivity (Friston, 1994; Lee et al., 2003). In order to establish neural integration as defined by IIT, it is essential that effective connectivity be established, as it is the causal interactions between neural elements that are fundamental to generating an irreducible complex.

The phase synchronization of neural oscillations across a range of frequency bands has become the dominant model to explain how integration is achieved (Varela et al., 2001), as well as specific thalamocortical circuits regulating information transfer between disparate cortical regions (Guillery and Sherman, 2002). Phase locking allows disparate cortical columns to engage in synchronized causal interactions, and neural elements can exchange and share information via forced oscillations, resonant loops, and transient oscillatory coupling (Buzsaki, 2006). Synchronization can occur across a range of spatial scales; local integration might refer to synchronized activity within a single cortical column or within a tight cluster of functionally related columns. Large-scale integration can include the synchronization of neural assemblies separated by great distances, between lobes or across hemispheres. The dense and reciprocal connections between thalamocortical columns enable a large number of neural elements to synchronize within a few hundred milliseconds to generate a unified, integrated neuronal assembly (Tononi et al., 1998; Tononi and Edelman, 2000). Oscillations in the gamma range (30–100 Hz) have received the most attention with regards to perceptual integration (Varela et al., 2001; Merker, 2013; Burwick, 2014) and such oscillations coexist with perceptual binding during normal waking consciousness (Gray and Singer, 1989; Joliot et al., 1994) and during dreaming (Llinas and Ribary, 1993). In fact, the synchronization of gamma oscillations unconstrained by sensory input has been invoked as a model for hallucinations and perceptual aberrations that occur during psychosis and in certain psychedelic states (Behrendt, 2003; Behrendt and Young, 2004). Synchronous firing of fast-spiking inhibitory interneurons, which strongly inhibit cortical pyramidal cells, generates cortical gamma oscillations (Cardin et al., 2009). Pertinently, these oscillations are regulated by 5HT_1A_ and 5HT_2A_ receptors on both pyramidal cells and their associated fast-spiking interneurons (Puig et al., 2010). 5HT_1A_ activation is largely inhibitory on both types of cell and 5HT_2A_ activation is excitatory. By activating fast-spiking interneurons, 5HT_2A_ activation potentiates cortical gamma oscillations and their synchronization, whilst 5HT_1A_ activation suppresses them (Puig et al., 2010; Puig and Gulledge, 2011). As the classical psychedelics are partial agonists at the 5HT_2A_ receptor, it is tempting to suggest that the combination of pyramidal cell depolarization and the promotion of gamma oscillations might explain many of the perceptual effects of these drugs. Increased sensitivity to incoming sensory data coupled with the generation of highly coherent gamma oscillations less constrained by external sensory inputs might result in the perceptual distortions, illusions and even hallucinations observed in the psychedelic state. Furthermore, as gamma oscillations are closely associated with neural integration, it is reasonable to surmise that the psychedelic state might be characterized by an increase in integration compared to a normal waking state. Although limited to measuring temporal correlations, coherence analysis of EEG and MEG traces is often used to quantify neural integration and studies exist that employed this technology to measure the effect of psychedelic drugs, including psilocybin, LSD, and ayahuasca (containing N,N-dimethyltryptamine, DMT) on brain function. However, the effect of these drugs on coherence is not consistent across all studies. A small study employing quantitative EEG (QEEG) to measure changes in oscillatory power and coherence following ayahuasca ingestion observed a highly integrated brain state (Stuckey et al., 2005). Dramatic increases in gamma coherence were widely distributed across the cortex, but especially over the occipital lobe (Stuckey et al., 2005). This is consistent with an earlier study showing an increase in power in the gamma band in ayahuasca users (Don et al., 1998). However, this result contrasts with other studies that found generalized decreases in power across all frequency bands (Riba et al., 2002, 2004).

A more recent psilocybin MEG study (Muthukumaraswamy et al., 2013) suggested that the psychedelic state is one of “dis-integrated” neural activity, with reduction in both oscillatory power and synchronization observed across all frequency bands. The authors suggest that this desynchronization results from 5HT_2A_-mediated excitation of deep layer pyramidal cells. However, it is noteworthy that the fMRI functional connectivity motifs unique to the post-psilocybin state were the most highly connected possible (Tagliazucchi et al., 2014). A disintegration of DMN integrity does not necessarily imply a reduction in overall neural integration and, indeed, an increase in between-network functional connectivity was consistently observed post-psilocybin (Roseman et al., 2014). This is further supported by a later network analysis of the psilocybin fMRI functional connectivity data, which shows the post-psilocybin state to be characterized by an increase in the integration between cortical areas (Petri et al., 2014). Together, this data suggests that psilocybin disrupts the *organization* of neural integration, but the overall effect appears to be an *increase* in the degree of integration.

Although this data is highly suggestive, the major limitation of functional connectivity and coherence analyses with regards to neural integration is that these temporal correlations do not necessarily imply effective connectivity. For example, propofol-induced anesthesia is associated with hypersynchronous oscillations in the alpha band (Supp et al., 2011), and yet effective connectivity, and thus integration as defined by IIT, is markedly reduced (Schroter et al., 2012; Casali et al., 2013; Gomez et al., 2013). Also, inferring causal interactions from functional imaging data requires additional techniques (Lee et al., 2003), including cortical perturbation approaches, discussed below. The currently available functional imaging data falls short of providing a definitive account of the effect of psychedelic drugs on neural integration across the brain. If the psychedelic state is to be understood as either a state of heightened consciousness or, conversely, a state of lower consciousness that lies between wakefulness and sleep, then techniques for measuring both information and integration concurrently must be considered.

### Measurement of integrated information in the psychedelic state

Although the cause-effect information specified by individual mechanisms should be lower in the psychedelic state compared to normal waking consciousness, concomitant with increased neural entropy and reduced differentiation between network states, the psychedelic state is not associated with any experiential drop in consciousness. In fact, most individuals feel a heightened sense of alertness and awareness under the influence of a psychedelic drug, rather than feeling that their consciousness is fading (Carhart-Harris et al., 2012). However, this is not a particularly reliable indicator of the effect of the drug on Φ and it isn't trivial to predict how integrated information might change when entering the psychedelic state from normal waking consciousness. As Φ is identified with the quantity of consciousness, an approximate measure of the effect of psychedelic drugs on this value would be extremely informative, revealing whether the psychedelic state is truly a state of increased or decreased consciousness and perhaps differentiating between the effects of classic psychedelics, such as psilocybin, and psychedelic anesthetics, such as ketamine.

Transcranial magnetic stimulation (TMS) coupled with EEG can be used to measure the response of the brain to direct perturbation of targeted cortical regions. Crucially, this approach separates effective connectivity (causal interactions) from functional connectivity (temporal correlations) (Massimini et al., 2005). During wakefulness, TMS stimulation produces a complex (differentiated) pattern of activity that becomes widely distributed across interacting cortical areas (integrated). However, during NREM sleep, the activity generated by TMS remains localized to the stimulation area, indicating a loss of effective connectivity (integration) (Massimini et al., 2005; Esser et al., 2009). This effect is also observed in the loss of consciousness produced by general anesthetics (Ferrarelli et al., 2010). Specific indices of cortical effective connectivity—and thus integration—can be used to quantify the spread of the TMS stimulus response (Ferrarelli et al., 2010) and could be useful in measuring the effect of psychedelic drugs on neural integration.

The TMS-EEG technique has been used to develop the Perturbational Complexity Index (PCI), an empirical measure of integrated information designed to differentiate between levels of consciousness in both healthy individuals and those with severe neurological injury (Casali et al., 2013; Sarasso et al., 2014). The PCI is calculated using the algorithmic compressibility of the EEG data generated following TMS perturbation. If integration is low, the TMS response is spatially restricted and the PCI is low. Likewise, if the response is stereotypical and undifferentiated, the EEG data will be highly compressible and the PCI will also be low. However, if the TMS perturbation produces a complex spatiotemporal pattern of activity that spreads to a large set of integrated areas, then the data cannot be compressed and the resulting PCI is high. The PCI is thus a measure of both the informational content and the integration of neural activity. Other entropy measures fail to differentiate between the information generated by the causal interactions of neural elements and random noise. This technique, however, only measures information resulting from deterministic interactions between neural groups. The PCI is successful in distinguishing between wakefulness and unconsciousness, both during deep sleep and anesthesia, and appears to be sensitive to graded changes in the level of consciousness (Casali et al., 2013). As such, it is conceivable that it could detect any difference in the level of consciousness between normal wakefulness and the psychedelic state.

## Conclusions, predictions, and future work

The task of the brain in shaping conscious experience has previously been framed as a problem of minimizing entropy whilst maintaining cognitive flexibility (Carhart-Harris et al., 2014). By examining the psychedelic state from the perspective of IIT, we have been able to equivalently restate the problem as the optimization of cause-effect information and cognitive flexibility. Psychedelic drugs increase cognitive flexibility (enhancing creativity, novel thinking, and imagination) but sacrifice a degree of cause-effect information specified by each mechanism (concept) within the conscious complex; each mechanism constrains the past and future states of the brain less stringently. This is a novel insight, as it contrasts with a simple Shannon interpretation of entropy increase, which would equate it with an increase in information. A decrease in information makes more sense, as it explains why the psychedelic state, although being a state of “expanded awareness,” might be non-optimal in terms of organizing and constraining cognition, categorizing, and differentiating concepts, and generally making sense of the progression of conscious moments. Furthermore, using this IIT-based model, it is possible to move beyond neural correlates and predict specific phenomenological features of the psychedelic state (Table 1).

**Table 1:** **Phenomenological features of the psychedelic state and their IIT correlates**.

**IIT Correlate**	**Phenomenological Features**
Increased entropy of cause-effect repertoires, moving closer to unconstrained past-future repertoires	Conscious experience is more fluid, dynamic, and unconstrained
Number of non-zero probability states in cause-effect repertoires increases	Meaning of concepts is altered, acquiring additional characteristics. Some concepts become associated with others in unusual ways because of enhanced conceptual overlap. Imagination and creativity is enhanced
ϕ-value of mechanisms is altered. ϕ-value of some mechanisms may increase from zero	The relative prominence of concepts in the experience changes. Some concepts become less noticeable, whilst others “pop out.” Completely novel concepts may also appear
Dimensionality of qualia space increased	Conscious experience seems richer and there is a sense of expanded awareness
Decrease in cause-effect information specified by individual mechanisms	Although awareness is expanded, there is a struggle to organize thoughts, to focus/concentrate, solve problems, and make sense of the world and the progression of conscious moments in the normal way

Despite reducing cause-effect information, from an experiential perspective, there is no indication that the classic psychedelics reduce the quantity of consciousness. In fact, quite the opposite appears to be the case—consciousness seems enhanced or elevated. Although this is not a reliable indicator as to the effect on Φ, which must be measured empirically, a tentative prediction can be made: any drug that increases neural entropy will produce a psychedelic state by expanding the number of states within the cause-effect repertoires of individual mechanisms, reducing the differentiation between network states and resulting in a state of unconstrained cognition and expanded awareness. However, the classic psychedelics (such as psilocybin) are able to maintain neural integration, and thus Φ and consciousness, whilst reducing cause-effect information. Although functional imaging data strongly supports this idea, it should be possible to test this more definitively using a TMS-EEG methodology. Psychedelic anesthetics, such as ketamine, whilst elevating neural entropy and so generating a form of psychedelic state, may lack the ability to maintain neural integration and, as with other anesthetics, lead to a breakdown of effective connectivity and unconsciousness in high doses (Ferrarelli et al., 2010). Again, this distinction ought to be measurable using TMS-EEG and other functional neuroimaging techniques.

## Funding

This work was supported in part by a funding from the Computational Neuroscience Unit of the Okinawa Institute of Science and Technology Graduate University.

### Conflict of interest statement

The author declares that the research was conducted in the absence of any commercial or financial relationships that could be construed as a potential conflict of interest.
